# Biomarkers of immune capacity, infection and inflammation are associated with poor outcome and mortality after stroke - the PREDICT study

**DOI:** 10.1186/s12883-019-1375-6

**Published:** 2019-07-03

**Authors:** A. Mengel, L. Ulm, B. Hotter, H. Harms, S. K. Piper, U. Grittner, J. Montaner, C. Meisel, A. Meisel, S. Hoffmann

**Affiliations:** 1 Department of Neurology Berlin, Charité Universitätsmedizin Berlin, Charitéplatz 1, 10115 Berlin, Germany; 2 Department of Neurology and Stroke, Universitätsklinik Tuebingen, Hoppe-Seyler-Str.3, 72076 Tuebingen, Germany; 3 Center for Clinical Research, The University of Queensland, Herston, Queensland 4029 Australia; 4 Center for Stroke Research Berlin, Charité Universitätsmedizin Berlin, Charitéplatz 1, 10115 Berlin, Germany; 5 Institute of Biometry and Clinical Epidemiology, Charité - Universitätsmedizin Berlin, corporate member of Freie Universität Berlin, Humboldt-Universität zu Berlin, Berlin, Germany; 6 Berlin Institute of Health, Charitéplatz 1, D-10117 Berlin, Germany; 7 Berlin Institute of Health (BIH), Anna-Louisa-Karsch 2, 10178 Berlin, Germany; 8 Neurovascular Research Laboratory, Vall d’Hebron Institut de Recerca, Passeig Vall d’Hebron 119-129, 08035 Barcelona, Spain; 9 NeuroCure Clinical Research Center Berlin, Charité Universitätsmedizin Berlin, Charitéplatz 1, 10115 Berlin, Germany

**Keywords:** Ischemic stroke, Biomarkers, Mortality, Outcome

## Abstract

**Background:**

Almost 40% of stroke patients have a poor outcome at 3 months after the index event. Predictors for stroke outcome in the early acute phase may help to tailor stroke treatment. Infection and inflammation are considered to influence stroke outcome.

**Methods:**

In a prospective multicenter study in Germany and Spain, including 486 patients with acute ischemic stroke, we used multivariable regression analysis to investigate the association of poor outcome with monocytic HLA-DR (mHLA-DR) expression, interleukin 6 (IL-6), interleukin 10 (IL-10), tumor necrosis factor alpha (TNF-alpha) and lipopolysaccharide-binding protein (LBP) as markers for immunodepression, inflammation and infection. Outcome was assessed at 3 months after stroke via a structured telephone interview using the modified Rankin Scale (mRS). Poor outcome was defined as a mRS score of 3 or higher which included death. Furthermore, a time-to-event analysis for death within 3 months was performed.

**Results:**

Three-month outcome data was available for 391 patients. Female sex, older age, diabetes mellitus, atrial fibrillation, stroke-associated pneumonia (SAP) and higher National Institute of Health Stroke Scale (NIHSS) score as well as lower mHLA-DR levels, higher IL-6 and LBP-levels at day 1 were associated with poor outcome at 3 months in bivariate analysis. Furthermore, multivariable analysis revealed that lower mHLA-DR expression was associated with poor outcome.

Female sex, older age, atrial fibrillation, SAP, higher NIHSS score, lower mHLA-DR expression and higher IL-6 levels were associated with shorter survival time in bivariate analysis. In multivariable analysis, SAP and higher IL-6 levels on day 1 were associated with shorter survival time.

**Conclusions:**

SAP, lower mHLA-DR-expression and higher IL-6 levels on day one are associated with poor outcome and shorter survival time at 3 months after stroke onset.

**Trial registration:**

www.clinicaltrials.gov, NCT01079728, March 3, 2010.

## Background

Many stroke patients experience severe consequences, with more than 40% of patients in the population suffering from disability, institutionalisation or even death at 3 months after the index event [1]. An early risk assessment of stroke outcome could help to optimize stroke care allocation in order to improve outcome [2], and to predict the patient’s individual prognosis. However, reliably predicting stroke outcome remains challenging. Several attempts to identify characteristics associated with poor outcomes have been made. A consistent association with poor outcome was found for the clinical factors age and stroke severity [3–5], as well as for post-stroke complications such as stroke-associated pneumonia (SAP) [6]. Further interesting targets for investigation have been blood-based biomarkers. It has been shown that several biomarkers are associated with poor outcome [7]. However, the added value of biomarkers over clinical variables with regard to prognosis remains controversial [8, 9]. In relation to the patient’s living will, biomarkers could help us to predict individual poor outcomes as early as possible in order to prevent pain and inefficient treatment. The PREDICT study was initially designed to better understand the underlying pathophysiology leading to SAP. We were able to demonstrate that a key biomarker of stroke-induced immunodepression syndrome (SIDS), mHLA-DR, was associated with SAP independently of dysphagia as the main risk for aspiration pneumonia [10]. By analysing the secondary end points of the PREDICT study, we wanted to evaluate and quantify the association of biomarkers of SIDS with poor outcome at 3 months after stroke which might serve to build more reliable prediction models for stroke outcome in the future.

## Methods

The methods used in this study (especially assessment of clinical and immunological data) have been described previously in the primary analysis of the PREDICT study (www.clinicaltrials.gov, NCT01079728, March 3, 2010) [10]. In short, the PREDICT study was a prospective, international, multicenter study with 11 participating study sites in Germany and Spain designed to better understand the underlying pathophysiology leading to SAP [10].

The study was approved by the ethics committees of the Charité- Universitätsmedizin Berlin (EA1/216/09), and of Hospital Vall d’Hebron Barcelona (No. 199 25/05/2012). All patients or their legal representatives gave written informed consent in accordance with the Declaration of Helsinki. The four inclusion criteria were: clinical diagnosis of acute ischemic stroke (NIHSS≥1) with any localization (anterior and posterior circulation) and with any recurrence rate (first-ever and recurrent stroke); symptom onset ≤36 h, and age ≥ 18 years [10]. During the study, the diagnosis was radiologically confirmed. Exclusion criteria were preexisting dysphagia, mechanical ventilation, antibiotic treatment or immunosuppressive therapy within the last 4 weeks and clinical or para-clinical signs of infection at study inclusion. Overall, 486 patients were recruited between January 2010 and December 2012. Blood samples were obtained within 36 h after symptom onset (day 1) and repeated on day 2 to 4. With the focus on early outcome we restricted the analysis to blood samples taken on day 1 after stroke.

### Clinical data

Clinically we documented socio-demographics, neurological deficits, comorbidities and stroke severity (NIHSS) on admission, as well as complications, and processes and outcomes during hospitalization [10]. We used the patient characteristics age at cerebrovascular event and sex in this analysis. In addition, we included the following comorbidities: hypertension (any existing treatment; patient self-reported; or new diagnosis during hospital admission); diabetes mellitus (any existing treatment; patient self-reported; or new diagnosis during hospital admission); previous stroke (neurological deficit more than 24 h prior to current event); and atrial fibrillation (patient self-reported or documented by standard ECG or Holter monitor). We also included in the analysis stroke severity on admission assessed by the NIHSS. This was categorized based on commonly defined cut-off points [11] into: mild impairment (NIHSS< 5), moderately severe impairment (NIHSS 5 to 15) and severe or very severe (NIHSS≥16) impairment [10]. Signs of pneumonia were clinically assessed on day 1 to 4 and on day 7 (or on the day of discharge in cases where the hospital stay was less than 7 days). During hospitalisation, diagnosis of SAP was made by the study or treating physician based on one or more of the following: clinical symptoms, suggestive clinical examination, radiological findings, and pathogen detection of pulmonary infection.

### Immunological data

Blood samples were obtained as soon as possible, but no longer than 36 h after symptom onset (day 1) and independent of the patient’s fasting status. They were analysed for immune (mHLA-DR, TNF-alpha) and infection (IL6, IL-10, LBP) parameters. For plasma collection, samples were centrifuged (200 g, 4 °C, 10 min) and stored at − 80 °C. Plasma concentrations of IL-6 were determined with the IMMULITE™ semi-automatic chemiluminescent immunoassay (Siemens Medical Solutions, Bad Nauheim, Germany). The detection limit for IL-6 was 2.0 pg/ml. The detection limit for IL-10 was 5.0 pg/ml. Based on visual binning, IL-10 was categorized into three categories: ≤5.0 pg/ml, 5.1–7.0 pg/ml and > 7.0 pg/ml. Expression of mHLA-DR on monocytes was determined by flow cytometry using a highly standardized quantitative assay as described earlier [12]. In short, whole blood in a Vacutainer tube (Becton Dickinson Biosciences, San Jose, CA, USA) containing EDTA was stained with 30 μl of monoclonal phycoerythrin-conjugated anti–HLA-DR and PerCP-Cy5.5–conjugated anti-CD14 antibodies (Quantibrite; Becton Dickinson Biosciences) in the dark at room temperature for 30 min. Erythrocyte lysis was achieved with 0.5 ml of lysing solution (Becton Dickinson Biosciences) for another 30 min at room temperature. Finally, the cells were washed with 1 ml of fluorescence activated cell sorting (FACS) buffer, and then analyzed on a FACSCalibur cytometer (Becton Dickinson Biosciences) to assess HLA-DR surface expression [10].

### Assessment of outcome

At 3 months after stroke, an outcome assessment was performed with each patient via a structured telephone interview assessing the mRS [13]. In line with other publications [3, 8], we defined poor outcome a priori as mRS score of 3 or higher (i.e. patients are moderately to severely disabled and need assistance with Activities of Daily Living, or have died). Furthermore, the cumulative proportion of deaths at 90 days post-stroke was calculated. If the patient or the indicated contact person (usually next of kin) were not traceable, vital status and, if available, the new address of the patient, were requested at the registration office.

### Statistics

All analyses were restricted to patients without missing values in the specific measures. The t-test was used to test differences among outcome groups in non-skewed continuous variables, and the Mann-Whitney test was used for skewed continuous variables. Pearson Chi-square test was used to test differences in proportions. Bivariate associations between dichotomous outcome categories (poor outcome at 3 month: mRS ≥ 3) and ordinal parameters were analyzed by the linear trend test. To estimate odds ratios (OR) and the corresponding 95% confidence intervals (CI) of the association between clinical variables and biomarkers with risk of poor outcome, logistic regression analysis was performed. As all patients with pneumonia were in the group of poor outcome, the problem of complete separation appeared. We applied a random effects logistic regression (random intercept for centers) with a Bayesian approach to account for heterogeneity between the participating centers. An informative prior for pneumonia (informed by a firth regression model with penalized likelihood estimation, prior: mean: 1, variance: 2.5) was applied (stata commands: bayes and melogit). Skewed variables were log-transformed or dichotomized before entering the analysis. ORs and 95% credible intervals are given. Using the Bayesian approach, it was possible to account for the complete separation of the data (Burn-in: 10.000, MCM sample size: 100.000, non-informative priors for all other variables except pneumonia with mean: 0 and variance: 10.000).

In the multivariable logistic regression analysis with random intercept for centers we included all relevant variables (*p* < 0.2 in bivariate analysis, or clinically relevant parameters). We additionally checked the association of logit (p = non-favorable outcome) with each variable using scatter plots to confirm whether a linear form was appropriate in the model.

Cox proportional hazards analysis and log-rank test (Mantel Cox) were used for survival analyses over the entire follow-up period of 3 months. Inspection of the scaled Schoenfeld residuals and formal testing of the proportional hazards assumption revealed that there was no substantial violation of the proportional hazards assumption.

For bivariate analysis, the survival function was estimated by the Kaplan-Meier (KM) method (product-limit estimator) and reported as cumulative proportion of deaths at 90 days after stroke with confidence interval and *p*-value of the log-rank test. Here non-categorized continuous variables were split around their median.

Finally, multivariable stratified Cox regression analysis was performed with all relevant parameters. Stratification was done with regard to centers. Hazard ratios (HR) with 95% CI and R-square measures are presented for the final model. In order to estimate the added benefit of the used biomarkers for survival at 3 months after stroke, a ROC-Curve analysis was performed for the final Cox regression models with and without biomarkers (command in STATA: roccomp).

All tests were two-tailed, and statistical significance was determined at an alpha level of 0.05. No adjustment for multiple testing was applied. Statistical analyses were performed with the SPSS 22.0 (IBM, Amonk, NY, USA), and Stata/IC 15.0.

## Results

### Patient demographic and clinical characteristics

Overall, 486 patients were recruited to the PREDICT study [10]. Two patients dropped out (one screening failure, one withdrew informed consent). Functional outcome or information on vital status at 3 months was available for 391 patients (80%), and only those were included in the functional outcome analysis. The mean age of our sample was 70 years; 42.2% were female; and median NIHSS at stroke onset was 4 points (inter-quartile range IQR: 2–7). Table 1 shows the demographic and clinical characteristics for patients stratified by good and poor functional outcome at 3 months. For survival analysis, 437 patients were included, while 47 either had missing values in an independent variable or were censored before the first event.

**Table 1:** Baseline characteristics stratified according to outcome after 3 months^e^

Characteristic	All	mRS 0–2	mRS 3–6	*p*-value
n (%)	391	231	160	
Female Sex, n (%)	165 (42.2)	83 (35.9)	82 (51.3)	0.003 ^a^
Age, yrs., mean (SD)	70 (12)	67 (12)	75 (10)	< 0.001 ^b^
NIHSS categories, n (%)12 missing				< 0.001 ^d^
0–4	217 (57.3)	156 (69.6)	61 (39.4)	
5–15	140 (36.9)	64 (28.6)	76 (49.0)	
16+	22 (5.8)	4 (1.8)	18 (11.6)	
Pneumonia, n (%)	20 (5.1)	0 (0)	20 (12.5)	< 0.001 ^a^
Comorbidities, n (%)				
Hypertension (13 missing)	313 (82.8)	179 (79.9)	134 (87.0)	0.072 ^a^
Diabetes mellitus	98 (26.3)	45 (20.5)	53 (34.6)	0.002 ^a^
Atrial Fibrillation (18 missing)	113 (30.3)	53 (24.1)	60 (39.2)	0.002 ^a^
Hypercholesterolemia (18 missing)	192 (51.5)	112 (50.2)	80 (53.3)	0.556 ^a^
Previous stroke (18 missing)	75 (20.1)	41 (18.6)	34 (22.2)	0.395 ^a^
mHLA-DR day 1 (pg/ml)				
Median (IQR) (4 missing)	16,422 [12268–21,990]	17,871 [13703–232,270]	15,062 [11340–19,333]	< 0.001 ^c^
IL-6 day 1 (pg/ml)				
Median (IQR)(10 missing)	4.3 [2.3–8.5]	3.6 [2.0–6.8]	5.5 [3.4–11.4]	< 0.001 ^c^
IL-10 day 1 (pg/ml)				
Median (IQR) (12 missing)	5.0 [5.0–5.0]	5.0 [5.0–5.0]	5.0 [5.0–5.0]	0.113 ^c^
LBP day 1 (pg/ml)				
Median (IQR) (11 missing)	7.5 [5.8–9.5]	7.0 [5.5–8.8]	8.2 [6.3–10.9]	< 0.001 ^c^
TNF-α day 1 (pg/ml)				
Median (IQR) (16 missing)	631 [459–887]	609 [452–868]	673 [462–898]	0.343 ^c^

^a^Pearson Chi-square test, ^b^ t-Test for unequal variance, ^c^ Mann-Whitney Test, ^d^ Chi-square test linear by linear

^e^Analyses were restricted to patients without missing values for mRS after 3 months or death within the study period and the respective variable

IQR = Q2-Q3

### Characteristics associated with poor 3-month outcome

Bivariate analysis showed that patients of older age, female gender and with higher NIHSS scores, SAP, diabetes or atrial fibrillation were more likely to have a poor outcome (mRS ≥ 3). Furthermore, lower mHLA-DR levels and higher IL-6 and LBP-levels at day 1 were associated with poor outcome at 3 months. Levels of IL-10 and TNF-alpha on day 1 had no influence on outcome.

Multivariable logistic regression with random intercept for centers showed that older age, diabetes mellitus, higher stroke severity (as measured by the NIHSS), SAP and lower mHLA-DR-expression were associated with poor outcome (Table 2).

**Table 2:** Characteristics associated with poor outcome (mRS 3–6): OR 95% credible intervals of final multivariable mixed logistic regression^a^ (*n* = 357), using Bayesian approach with informative prior for pneumonia

Characteristic	OR	95% CI
Female sex	1.47	0.80–2.42
Age (yrs)	1.07	1.04–1.10
Pneumonia	50.34	5.38–230.85
NIHSS categories		
0–4	1.00	
5–15	3.01	1.94–5.02
16+	14.43	5.09–33.92
Diabetes mellitus	3.21	1.75–5.32
Hypertension	0.90	0.37–1.86
Atrial fibrillation	0.72	0.40–1.19
Biomarker		
mHLA-DR day 1^a^ (pg/ml)	0.42	0.28–0.59
LBP day 1^a^ (pg/ml)	1.64	0.89–2.85
IL6^a^ (pg/ml)	1.19	0.88–1.59
IL10 (> 5) (pg/ml)	1.48	0.76–2.72

Analysis was restricted to patients without missing values for all variables including mRS at 3 month (N = 357). Logistic mixed regression was performed using a Bayesian approach and an informative prior for pneumonia. (informed by Firth logit model without random effects for centers: normal prior with mean 1 and variance 2.5), MCMC sample size: 100.000, Burn in: 10.000)

^a^Skewed variables were log transformed before entering the analysis, OR and 95% credible interval are given for the log transformed values

### Characteristics associated with mortality

In bivariate analyses (Table 3) female sex, older age, higher NIHSS score, SAP, atrial fibrillation, lower mHLA-DR expression as well as higher IL-6-levels on day 1 were associated with shorter survival time. Similar to a lack of association with poor outcome, IL-10 levels and TNF-alpha levels on day 1 were not associated with an increased risk of mortality.

**Table 3:** Bivariate survival analysis: Cumulative proportion of deaths at 3 month after stroke ^a^

Characteristic *N* = 484 Death (N = 21)	Cumulative Proportion (%) of deaths (95% CI)	*p*-value
Overall	4.8 (2.6–7.0)	
Sex		0.018
female (*n* = 209)	7.8 (3.7–12.0)	
male (*n* = 274)	2.6 (0.4–4.8)	
Age, yrs		< 0.001
< 64 (*n* = 148)	2,8 (0–5,9)	
65–74 (*n* = 154)	1.6 (0–3.8)	
75–84 (*n* = 137)	6.8 (2.3–11.3)	
85+ (*n* = 45)	17.7 (5.7–29.6)	
NIHSS		< 0.001
0–4 (*n* = 265)	2.3 (0.3–4.3)	
5–15 (*n* = 178)	7.2 (2.9–11.3)	
16+ (n = 27)	22.3 (5.1–39.5)	
Pneumonia		
Yes (*n* = 25)	28.9 (9.1–48.7)	< 0.001
No (*n* = 454)	3.5 (1.5–5.5)	
Comorbidities		
Hypertension		0.785
Yes (*n* = 388)	5.3 (2.8–9.1)	
No (*n* = 75)	3.2 (0–7.5)	
Diabetes mellitus		0.523
Yes (*n* = 128)	4.1 (0.2–8.0)	
No (*n* = 330)	5.5 (2.8–8.2)	
Atrial Fibrillation		0.013
Yes (*n* = 138)	9.6 (4.1–15.1)	
No (*n* = 319)	4.6 (0.8–5.1)	
Hypercholesterolemia		0.778
Yes (*n* = 237)	5.7 (2.4–9.0)	
No (*n* = 219)	4.3 (1.4–7.2)	
Previous stroke		0.276
Yes (*n* = 99)	6.6 (0.9–12.3)	
No (*n* = 359)	4.6 (2.2–6.9)	
Biomarkers		
mHLA-DR day1		0.014
< =16,422 (*n* = 234)	7.1 (3.6–10.6)	
> 16,422 (n = 237)	2.6 (0.2–5.0)	
IL-6 day1		0.023
< =4.3(n = 234)	1.6 (0–3.4)	
> 4.3 (*n* = 231)	6.2 (2.9–9.5)	
IL-10 day1		0.459
<=5.0 (*n* = 387)	3.4 (1.4–5.4)	
>5.0 (*n* = 76)	6.2 (0.3–12.1)	
LBP day1		0.073
<= 7.5 (*n* = 233)	1.6 (0–3.6)	
>7.5 (n = 231)	6.3 (2.7–9.8)	
TNF-α day1		0.216
<=631 (*n* = 214)	2.1 (0–4.3)	
>631 (*n* = 244)	5.8 (2.5–9.1)	

^a^Analyses were restricted to patients without missing values in the respective ^#^cumulative Proportion of Deaths is calculated as 1- the Kaplan-Meier estimator

Multivariable Cox regression adjusted for clustering by centers revealed SAP and higher IL6-levels on day 1 as being associated with shorter survival time in the 3 months after stroke onset (Table 4). Adding biomarkers on day 1 to the model significantly improved the model fit for survival in the 90 days of follow-up over a model with clinical variables alone (Table 4, Likelihood Ratio-Test: *p* = 0.037).

**Table 4:** Characteristics associated with mortality in patients with acute stroke during 3-months follow-up. HR (hazard ratio). The final model included *n* = 434 complete cases and 16 events. Median follow-up time was 94 days IQR: 90–101 days)^a^. Nagelkerke *R*^2^ = 0.18

Characteristic	HR	95% CI	p-value
Female sex	1.91	0.60–6.10	0.275
Age, yrs.	1.03	0.97–1.10	0.288
NIHSS categories			
0–4	1.0		
5–15	0.82	0.22–3.10	0.765
16+	1.39	0.25–7.70	0.705
Pneumonia	5.43	1.22–24.11	0.026
Diabetes mellitus	0.74	0.19–2.88	0.660
Hypertension	0.63	0.15–2.88	0.529
Atrial Fibrillation	1.44	0.46–4.49	0.529
Biomarker			
LBP day 1^b^ (pg/ml)	2.26	0.65–7.90	0.288
mHLA-DR^b^ (pg/ml)	0.53	0.13–2.07	0.359
IL6^b^ (pg/ml)	1.97	1.05–3.70	0.034
IL10 (> 5) (pg/ml)	1.02	0.27–3.81	0.979
AUC survival Cox regression model without biomarkers at day 1			0.76 (0.61–0.90)
AUC survival Cox regression model with biomarkers at day 1			0.81 (0.68–0.94)

^a^Analysis was restricted to patients without missing values for the respective variables (*N* = 437). Variables were eliminated using backward elimination procedure, stepwise excluding all variables with *p* > 0.200. AUCs were calculated with a cut-off in follow-up time at 90 days.434). Cox regression stratified by centers

^b^log-transformed

## Discussion

The prognostic value of blood biomarkers in patients with acute stroke could potentially be of great importance for clinical routine. In our study, lower mHLA-DR levels, and higher IL-6 and LBP levels on day 1 were associated with poor outcome (mRS ≥ 3) at 3 months. Furthermore, we found that lower mHLA-DR levels were associated with poor outcome at 3 months after adjustment for clinical characteristics and other relevant biomarkers. ROC analysis of the Cox regression models showed that including the biomarkers on day 1 improved the area under the curve compared to a Cox regression model using clinical data alone (age, sex, NIHSS> 16, SAP, diabetes mellitus, hypertension, atrial fibrillation).

In line with previous studies [14, 15] we found that higher mHLA-DR was inversely associated with the odds of having poor outcome. Monocytes are of paramount immunological importance due to their contribution firstly to adaptive immunity as antigen-presenting cells, and secondly to innate immunity through the expression of pattern recognition receptors [14]. Impaired monocyte function results in insufficient antigen-presentation and decreased expression of secreted or membrane-bound costimulatory molecules, and it therefore may contribute to reduced lymphocyte responses [16]. HLA-DR has previously been identified as a prototypical biomarker for stroke-induced immunodepression [12].

IL-6 has been widely investigated as biomarker for early inflammation. Higher IL-6 levels after stroke have been associated with poor outcome, but the additional value over clinical data was moderate [17]. To the best of our knowledge, this is the first study that suggests an association between IL-6 (baseline) and death in stroke patients. Among lacunar stroke patients, IL-6 and TNF receptor concentrations were associated with risk of recurrent vascular events and with the effect of antiplatelet therapies [18]. In patients with atrial fibrillation, increased IL-6 seems to be related to higher risk for both stroke and major bleeding, as well as to thromboembolic events and vascular death [19]. However, a meta-analysis of 24 studies indicated that the translation of IL-6 into clinical practice for prognosis of poor outcome after stroke was unlikely to be of benefit [17]. In our study, TNF-alpha was not substantially associated with outcome or mortality.

In in vitro models, IL-10 is known to mediate neuroprotection, but clinically its role as prognostic marker for outcome is controversial [20, 21]. In the present study we could not find a substantial association of IL-10 expression with outcome and mortality.

In our study higher LBP levels were - in univariate analysis - associated with poor outcome. LBP is an acute phase protein essential for the response to bacterial lipopolysaccharides, or endotoxin, in Gram-negative bacteria [22]. LBP may rise as a response to subclinical infections or even to intestinal translocations [23]. However, LBP may be an early post-stroke infection marker, especially for SAP, although the majority of patients with SAP were diagnosed on day 3 and the association with death was still present after adjustment for SAP. Recently, smaller studies which investigated LBP did not find a positive association for infection or poor outcome in stroke patients [24, 25].

Regarding clinical variables associated with poor outcome and mortality, our findings are in line with previous studies [26–29]. Older age, pneumonia and stroke severity are known to be associated with poor outcome and mortality. In our study, female sex and presence of diabetes mellitus were also independently associated with poor outcome. Our data concurs with previous studies reporting a less favourable outcome in women compared to men [30–32]. Possible explanations for sex differences in stroke outcome include women being older [33], having a decreased likelihood of receiving rt-PA treatment [34], and having a higher incidence of comorbidities (e.g. depression) [35]. Available research on the association of diabetes mellitus with outcome is inconclusive. Whereas previous studies had found a higher risk of mortality in diabetic patients [36, 37], more recent studies have not shown such an association, especially after adjusting for post-stroke hyperglycaemia [38].

In the management of critically ill patients, a sensitive and specific prognosis of outcome is desirable. Outcome prognosis (for example chances of recovery, expected length of recovery period, and anticipated quality of life) influences our treatment decisions about withholding or withdrawing life-sustaining therapies. However, the accuracy of early outcome prognosis by physicians or through using current scoring systems is only moderate. Therefore clinicians hope to improve prognosis of outcome by developing sensitive and specific biomarkers for clinical practice [39]. The consistent and strong association of the clinical variables age and stroke severity with poor outcome makes it difficult for biomarkers to significantly add information for prognostic models. Furthermore, a variety of different biomarkers have been implicated in stroke prognosis, yet to date no biomarker has found its translational way from bench to bedside [40]. Even though we recognize that biomarkers alone should not be decisive in clinical decision making, they do harbour the unique advantage of providing insights into the mechanisms leading to poor outcome and therefore may lead to new therapeutic strategies [41, 42].

We identify several limitations of our study: firstly, we had a relatively short follow-up period of 90 days after stroke onset. However, several studies have shown that short-term outcome is strongly associated with long-term outcome [43, 44], and that on the whole both show the same associations with clinical variables [4], as well as with biomarkers [8]. Secondly, in our study, the attrition rate between the recruitment of the 484 patients and follow-up at 3 months for mRS (via a structured telephone interview) was 20.5%. This rate and our follow-up method are comparable to those of other studies [45, 46]. Information on vital status was available for all patients. Thirdly, within the analysed cohort of patients, there is a likely selection bias towards better outcome since the severity of initial strokes (median NIHSS 4 points) was relatively low. Whereas the distribution of overall favourable and poor outcome is comparable to those of other studies [45, 46], the 4.9% mortality rate in our cohort was low. In order to achieve a representative cohort of stroke patients, we included patients with acute ischemic stroke of any severity (NIHSS≥1). The challenges of obtaining informed consent from stroke patients are widely acknowledged, with stroke severity being strongly associated with not obtaining informed consent from stroke patients [47, 48], as well as with mortality after stroke [4]. Though study inclusion of patients via “proxy consent” by their legal representative was possible, we found that this was often not feasible due to the relatively short time window of study recruitment within 36 h after symptoms onset. And lastly, we suggest that the subgroup analysis on mortality be interpreted with caution due to the low case number (*n* = 21). In spite of these limitations, the two promising candidates for future investigations are SAP as a potentially modifiable post-stroke complication, and IL-6 as blood-based biomarker, both of which were associated with shorter survival in our patient population.

## Conclusion

After combining with clinical variables (age, gender, stroke severity, comorbidities and stroke-associated pneumonia) we identified lower mHLA-DR expression in the acute phase of stroke as being associated with poor outcome at 3 months after the index event. In addition, IL-6 was associated with shorter survival, even after adjustment for clinical data. Both biomarkers could help to develop better prediction models for stroke outcome in the future.

## Data Availability

The datasets used and/or analysed during the current study available from the corresponding author on reasonable request.

## Abbreviations

AUC: Area under the curve measures
CI: Confidence intervals
FACS: Fluorescence activated cell sorting FACS
HR: Hazard ratios
IL-10: Interleukin 10
IL-6: Interleukin 6
IQR: Inter-quartile range
KM: Kaplan-Meier
LBP: Lipopolysaccharide-binding protein
mHLA-DR: monocytic HLA-DR
mRS: modified Rankin Scale
NIHSS: National Institute of Health Stroke Scale
OR: Odds ratio
SAP: Stroke-associated pneumonia
SIDS: Stroke-induced immunodepression syndrome
TIA: Transient ischemic attack
TNF-alpha: Tumor necrosis factor alpha
